# Relevance of JAK2V617F positivity to hematological diseases - survey of samples from a clinical genetics laboratory

**DOI:** 10.1186/1756-8722-4-4

**Published:** 2011-01-14

**Authors:** Wanming Zhao, Rufei Gao, Jiyun Lee, Shu Xing, Wanting T Ho, Xueqi Fu, Shibo Li, Zhizhuang J Zhao

**Affiliations:** 1 Department of Pathology, University of Oklahoma Health Sciences Center, Oklahoma City, Oklahoma 73104, USA; 2 Edmond H. Fischer Signal Transduction Laboratory, College of Life Sciences, Jilin University, Changchun, China; 3 Department of Pediatrics, University of Oklahoma Health Sciences Center, Oklahoma City, Oklahoma 73104, USA

## Abstract

**Background:**

JAK2V617F is found in the majority of patients with Ph- myeloproliferative neoplasms (MPNs) and has become a valuable marker for diagnosis of MPNs. However, it has also been found in many other hematological diseases, and some studies even detected the presence of JAK2V617F in normal blood samples. This casts doubt on the primary role of JAK2V617F in the pathogenesis of MPNs and its diagnostic value.

**Methods:**

In the present study, we analyzed JAK2V617F positivity with 232 normal blood samples and 2663 patient blood, bone marrow, and amniotic fluid specimens obtained from a clinical genetics laboratory by using a simple DNA extraction method and a sensitive nested allele-specific PCR strategy.

**Results:**

We found JAK2V617F present in the majority (78%) of MPN patients and in a small fraction (1.8-8.7%) of patients with other specific hematological diseases but not at all in normal healthy donors or patients with non-hematological diseases. We also revealed associations of JAK2V617F with novel as well as known chromosomal abnormalities.

**Conclusions:**

Our study suggests that JAK2V617F positivity is associated with specific hematological malignancies and is an excellent diagnostic marker for MPNs. The data also indicate that the nested allele-specific PCR method provides clinically relevant information and should be conducted for all cases suspected of having MPNs as well as for other related diseases.

## Background

Ph- myeloproliferative neoplasms (MPNs) represent a group of conditions including polycythemia vera (PV), essential thrombocythemia (ET), and primary myelofibrosis (PMF) [1]. The major molecular lesion in these diseases is JAK2V617F, which occurs in approximately 96% of PV, 65% of PMF, and 55% of ET cases [2-7]. Studies demonstrated that transgenic expression or knock-in of JAK2V617F caused MPN-like phenotype in mice [8-14]. JAK2V617F has thus become a valuable marker for diagnosis of MPNs and an excellent target for therapeutic drug development [15,16]. However, JAK2V617F has also been found in refractory anemia with ringed sideroblasts and thrombocytosis, in patients with Budd-Chiari syndrome, and in sporadic cases of other hematological diseases including leukemia and myelodysplastic syndrome (MDS) [15-17]. Interestingly, by using a sensitive allele-specific PCR approach, we screened over 4000 blood samples randomly collected from a Chinese hospital population and found nearly 1% of samples to be JAK2V617F positive, although few of them meet the criteria for diagnosis of MPNs [18]. Intriguingly, a study using a more sensitive method revealed the presence of JAK2V617F in around 10% of normal blood samples [19]. This casts doubt on the primary role of JAK2V617F in the pathogenesis of MPNs and its diagnostic value [17]. In order to more fully define the role of JAK2V617F in hematological diseases, the current study analyzed nearly 3000 blood and tissue specimens. We found JAK2V617F present in the majority of MPN patients and in a small fraction of patients with other specific hematological diseases but not at all in healthy donors or patients with non-hematological diseases. Our data also revealed associations of JAK2V617F with novel as well as known chromosomal abnormalities.

## Methods

### Sample collection and DNA extraction

The patient samples used in the current study were residual blood, bone marrow, and amniotic fluid products collected for routine fluorescence in situ hybridization and karyotype analysis done between 2003 and 2006 in the Genetics Laboratory, Department of Pediatrics at University of Oklahoma Health Sciences Center. De-identified normal blood samples were collected from health donors subjected to routine physical exams at local clinical laboratories. Institutional review board approval was obtained before these samples were analyzed. White blood cells from the above clinical samples were fixed with acetic acid/methanol (1:3) and stored in the same solution at -20°C. To isolate DNA for PCR analyses, the cells were pelleted by centrifugation, washed with 70% ethanol, and then resuspended in a buffer containing 100 mM Tris-HCl (pH 8.0), 1% (v/v) Tween 20, and 25 μg/ml proteinase K. After 2 hr incubation at 55°C, the samples were heat-treated at 95°C for 10 min to inactivate proteinase K. Then, they were directly used for detection of JAK2V617F by using a nested allele-specific PCR method as described below. For the JAK2V617F-positive samples identified by nested allele-specific PCR, DNAs were purified from the proteinase K digests by performing phenol/chloroform extractions. The purified DNAs were subjected to direct allele-specific PCR analyses without going through the initial PCR amplification step.

### PCR amplification and analysis of PCR products

JAK2V617F was detected by nested allele-specific PCR method as described previously [18]. Briefly, initial PCR amplifications were performed with two primers and 0.5 μl of cell lysates obtained above in a total volume of 20 μl for 35 cycles. For allele-specific PCR, 0.5 μl of the initial PCR product was used for further PCR amplification with allele-specific nested primers (a mixture of 4 primers) for 35 cycles. Taq DNA polymerase was used for both initial and nested PCR. The PCR products were resolved on 3% agarose gel, and DNA bands were visualized by staining with ethidium bromide. Gel images were captured by using the FluorChem SP imaging system from Alpha Innotech. Each JAK2V617F-positive sample was confirmed by performing the allele-specific PCR analyses with phenol/chloroform-purified DNA samples. To avoid possible cross-contaminations, control experiments with water replacing DNA samples were routinely performed.

### Statistical analysis

Statistical analyses were performed by using the GraphPad Prism program. Differences in JAK2V617F percentages and ages were accessed by Fisher's exact tests and *t *tests, respectively. P values of less than 0.05 (two tailed) are considered significantly different.

## Results and Discussion

**Figure 1:**
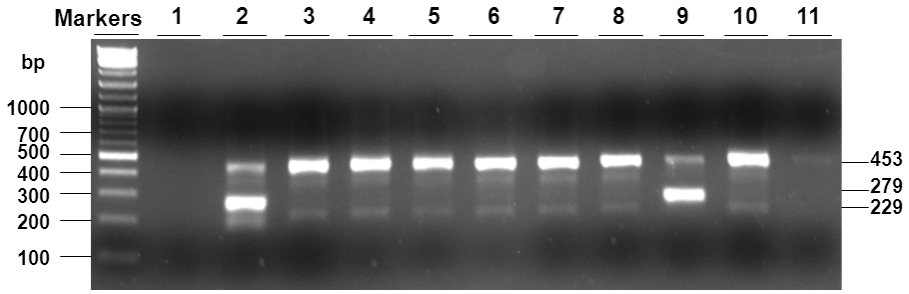
**Detection of JAK2V617F by allele-specific PCR**. Nested PCR was performed with crude genomic DNA samples as described in Methods. PCR products were analyzed on 3% agarose and visualized by ethidium bromide staining. The expected PCR products are 453 bp (for both JAK2V617F-positive and -negative alleles), 279 bp (for JAK2V617F-positive allele), and 229 bp (for JAK2V617F-negative allele). Lane 1 was done with water in place of genomic DNA samples to rule out possible cross-contaminations. Lane 11 did not give a clear PCR product and was excluded from further analysis. Samples 2 and 9 are JAK2V617F-positive, while all the rest are JAK2V617F-negative.

**Figure 2:**
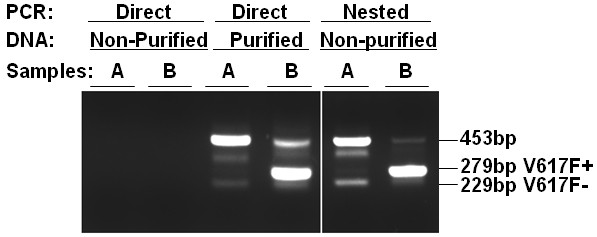
**Comparison of JAK2V617F detections by using direct and nested allele-specific PCR with non-purified and purified DNA samples**. Non-purified and phenol/chloroform extraction-purified DNAs from JAK2V617F-negative (lane A) and JAK2V617F-positive (lane B) samples were subjected to direct or nested allele-specific PCR analyses as indicated. The final PCR products were analyzed on 3% agarose and visualized with ethidium bromide staining. Note that the direct PCR analyses of purified DNAs and the nested PCR analyses of non-purified DNAs gave rise to consistent results while the direct PCR of non-purified DNAs did not yield any PCR product.

Figure 1 illustrates typical results of JAK2V617F detection by using nested allele-specific PCR. The conditions strongly favor the detection of the mutant allele with a sensitivity of about 0.25% JAK2V617F mutation rate according to our previous studies with standard DNAs [18]. To rule out possible cross-contaminations associated with nested PCR, control experiments were routinely performed with water instead of DNA samples. Of the roughly 3000 samples analyzed, a total of 2895 gave rise to PCR products, and 32 of these were identified as JAK2V617F positive. Samples that failed to give rise to clear PCR products were excluded from further analysis. For all the JAK2V617F-positive samples, DNAs were purified and enriched from the proteinase K digests by performing phenol/chloroform extractions. These purified DNAs were dissolved in a small volume of water to give rise to DNA concentrations ranging from 0.02 to 0.2 mg/ml. Upon direct allele-specific PCR analyses, they all gave rise to JAK2V617F-positive bands and thus confirmed the results of our initial screening with nested PCR. Figure 2 shows typical results of a JAK2V617F-positive sample together with a JAK2V617F-negative one. Note that direct analysis of non-purified/non-enriched samples with direct allele-specific PCR failed to produce any PCR product. Therefore, our nested allele-specific PCR analyses increase the sensitivity for detecting both JAK2V617F-positive and -negative samples with low DNA concentrations and poor quality.

Table 1 summarizes the data of our JAK2V617F analyses. We identified a total of 32 JAK2V617F-positive cases out of 665 patients with hematological diseases but not at all in 2230 samples from normal donors and patients with non-hematological diseases (P < 0.0001). Within the hematological diseases, the average age of JAK2V617F-positive patients was significantly higher than that of JAK2V617F-negative ones (P = 0.003). Among the 32 JAK2V617F positive samples, 14 were from MPN patients, representing 78% of total cases in the group. This is significantly higher than the percentages found in other groups analyzed in this study (P value < 0.0001). These MPN patients displayed clinical manifestations of polycythemia, thrombocytosis, and/or splenomegaly. The average age of these JAK2V617F-positive MPN patients was 69 (ranging from 48-85), which is consistent with the fact that MPNs mainly occur in older people. However, the ages of these JAK2V617F-positive patients were not significantly different from those of JAK2V617F-negative patients (P = 0.3). Of these 14 cases, all but three were shown to have a normal karyotype. Among the three patients with chromosomal abnormalities, the first had monosomy 20, the second lost chromosome Y, and the third displayed an isochromosome of the entire long arm of chromosome 8. Many reports have shown an association of monosomy 20 with primary myelofibrosis and a loss of the Y chromosome in male MPN patients [20]. However, to our knowledge, ours is the first case of isochromosome 8 in MPNs. Interestingly, two of the four JAK2V617F-negative MPN samples also had chromosome abnormalities; one lost chromosome Y, and the other had a translocation between chromosomes 9 and 12 at breakpoints near 9p21 and 12p12. Note that the JAK2 gene is located at 9p24.1. It would be interesting to know if the translocation affects the expression of JAK2. In all, the data suggests that cytogenetic analysis continues to provide useful information for the diagnosis and treatment of MPNs that cannot be obtained with JAK2V617F detection alone. Table 2 lists all the MNP- and JAK2V617F-positive cases with abnormal karyotypes.

**Table 1:** Results of JAK2V617F Tests

Sample Types and Diagnosis	Number of total samples	Number of V617F+ samples	Percentage of V617F+ samples	Average ages of V617F- samples	Average ages of V617F+ samples
**Blood and Bone Marrow Specimens**					
**Hematological diseases**	**665**	**32**	4.8*	**52.8**	64.6**
Ph- MPNs	18	14	78*	62.3	69.2
Leukocytosis	23	2	8.7*	50.8	62.5
Acute myeloid leukemia	113	6	5.3*	47.9	56.8
Leukemia (unspecified)	70	3	4.3*	47.1	66.6
Anemia	65	2	3.1*	54	76.8
Lymphoma	98	3	3.1*	55.6	60.2
MDS and probable MDS	111	2	1.8	55.8	49.9
Acute lymphoblastic leukemia	21	0	0	13	-
Chronic lymphocytic leukemia	14	0	0	67.4	-
Chronic myeloid leukemia	32	0	0	47.9	-
Multiple myeloma	50	0	0	65.1	-
Thrombocytopenia	29	0	0	60.1	-
Other hematological diseases	21	0	0	54.4	-
**Non-hematological diseases**	**1731**	**0**	**0**	**7.3**	-
Developmental disorders	1370	0	0	4.9	-
Multiple miscarriage/infertility	83	0	0	30.2	-
Others	278	0	0	14.8	-
**Normal samples**	**232**	**0**	**0**	**54.5**	-

**Amniotic Fluid Specimens**					
**Cytogenetic screening**	**267**	**0**	**0**	**31.3**	-

* P < 0.05 when comparing the percentage of JAK2V617F positivity with normal samples.

** P < 0.05 when comparing the ages of JAK2V617F+ and JAK2V617F- samples within each disease.

**Table 2:** Chromosomal Abnormalities in MPN and JAK2V617F-Positive Samples

Diagnosis	Cases	V617F	Chromosomal Abnormalities
Ph-MPNs	1	+	Monosomy 20
	1	+	Loss of chromosome Y
	1	+	Isochromosome of the entire long arm of chromosome 8
	1	-	Loss of chromosome Y
	1	-	Chromosomes 9 and 12 translocation at 9p21 and 12p12
Leukocytosis	1	+	Deletion of the long arm of chromosome 16 at 16q23

AML	1	+	Chromosomes 8 and 21 translocation 8q22 and 21q22
	2	+	5q deletion and monosomy 7
	1	+	Deletion of the long arm of chromosome 5 at 5q21, deletion of the short arm of chromosome 6 at 6p21.3, and monosomy 9

Leukemia (unspecified)	1	+	Trisomy 8
	1	+	Trisomy 20

Anemia	1	+	Trisomy 8

MDS	1	+	Deletion of the long arm of chromosome 5 at breakpoint 5q31

We also found a total of 18 JAK2V617F-positive cases out of 480 patients (38-81 years old) with leukocytosis, acute myeloid leukemia (AML), unspecified leukemia, anemia, and MDS. In contrast, we did not find a single JAK2V617F-positive case in blood samples from 232 healthy donors with comparable ages (ranging from 45 to 75 years). This suggests a strong association of JAK2V617F positivity with these hematological diseases (P value = 0.001). Note that the ages of these normal donors were not significantly different from those of healthy donors and that there was no significant difference in the ages of JAK2V617F-positive and -negative patients for each hematological disease. It should also be pointed out that leukocytosis and anemia do not necessarily represent specific diseases but rather manifestations of a number of hematological diseases. We do not have information regarding precise diagnosis for these patients. In addition, since about 10% of MPN patients eventually develop AML [21], some of the JAK2V617F positivity found in leukemia may be derived from MPNs. However, there was no evidence that any of these patients had a previous history of MPNs. Interestingly, more than half of JAK2V617F-positive patients had chromosomal abnormalities (see Table 2). One leukocytosis patient displayed a deletion of the long arm of chromosome 16 at breakpoint of 16q23, but this did not involve the CBFB gene that is frequently rearranged in AML-M4 [20]. One of the AMLs had a translocation between chromosomes 8 and 21 at breakpoints of 8q22 and 21q22, which is commonly associated with AML-M2 [20]. Rare cases of JAK2V617F positivity have recently been reported in AML-M2 patients [22]. Two other AML cases had 5q deletion and monosomy 7, which is frequently found in this disease [20]. Another case of AML had a deletion of the long arm of chromosome 5 at the breakpoint of 5q21, a deletion of the short arm of chromosome 6 at breakpoint of 6p21.3, and monosomy 9. Two of the three unspecified leukemia cases showed abnormal karyotypes, one with trisomy 8 and the other with trisomy 20. An extra chromosome 8 is frequently present in AML patients but trisomy 20 has not been found to be associated with any particular type of leukemia [20]. Two out of 65 anemia and two out of 111 MDS patients were found to be JAK2V617F positive. None of these four positive patients had a preceding MPN. One of the anemia patients had a normal karyotype, while the other had trisomy 8, suggesting that the anemia may be associated with MDS, which often has trisomy 8 [20]. One of the MDS patients had a deletion of the long arm of chromosome 5 at breakpoint 5q31.

We also analyzed a total of 98 lymphoma cases. Interestingly, three were found to be JAK2V617F positive, though all had a normal karyotype. JAKV617F-positive lymphoma cases were also found in our previous studies with the Chinese population [18]. The pathological significance of this finding, however, needs further investigation since JAK2V617F is not thought to affect lymphocytes [15,16].

Nonetheless, JAK2V617F appears to be limited to specific types of hematological diseases, since no JAK2V617F-positive cases were found in patients with acute lymphoblastic leukemia, chronic lymphocytic leukemia, chronic myeloid leukemia, multiple myeloma, or thrombocytopenia. The ages of these patients, except for those with acute lymphoblastic leukemia, were not significantly different from the ages of the patients described above. Furthermore, all blood or bone marrow samples from patients (n = 1731) with non-hematological diseases were JAK2V617F negative. The majority of these were from children who possibly have developmental disorders (e.g., Down syndrome, developmental delay, congenital heart defect, dysmorphic features, failure to thrive, etc.) due to congenital genetic defects. There are also a number of samples from adult patients with infertility or multiple miscarriages. Finally, we included 267 amniotic fluid samples in this study. These samples were from pregnant women of advanced maternal age and were originally collected to test possible genetic abnormality of the fetus. None of these samples showed any sign of JAK2V617F positivity.

Of the 2895 DNA samples available for analyses, 32 were JAK2V617F positive. These positive samples are predominantly present in MPN patients but also in a small fraction of patients with other hematological diseases including AML, anemia, MDS, and lymphoma. Positive samples were not found at all in health donors of comparable ages and individuals who did not have hematological diseases. This suggests JAK2V617F positivity is associated with specific hematological malignancies. This, however, does not contradict our previous data, which revealed the presence of JAK2V617F in many patients without a MPN phenotype but who had cerebral and cardiovascular disorders [18]. First, our current analysis covered a set of clinical samples very different from our previous study. Second, heart disease and stroke are often associated with blood abnormality, although they are not necessarily linked to malignant blood diseases. We believe the JAK2V617F-induced pre-MPN phenotype may increase the likelihood of other blood cell-related diseases. In any case, relevance of JAK2V617F positivity with vascular disorders deserves further investigations.

## Conclusions

Our data demonstrate that JAK2V617F is predominantly present in MPN patients and is associated with specific hematological malignancies (P < 0.05). Our current data also suggest the nested allele-specific PCR method is sensitive enough to provide clinically relevant information but not so sensitive as to give false or misleading information [17]. With a sensitivity of about 0.25% mutation rate, the method is simple, quick, and inexpensive [18]. It requires a very small amount of DNA, and even non-purified DNA of poor quality can be successfully analyzed. For these reasons, this test should be conducted on all cases suspected of having MPNs as well as on other related diseases.

## List of abbreviations

AML: acute myeloid leukemia; ET: essential thrombocythemia; MDS: myelodysplastic syndrome; MPN: myeloproliferative neoplasm; PMF: primary myelofibrosis; PV: polycythemia vera.

## Competing interests

The authors declare that they have no competing interests.

## Authors' contributions

WZ, RG, JL, SX, and WTH conducted the research experiments; XF and SL designed the experiments; ZJZ designed the experiments and wrote the manuscript. All authors read and approved the final manuscript.
